# First Isolation of *Rickettsia slovaca* from a Patient, France

**DOI:** 10.3201/eid0901.020192

**Published:** 2003-01

**Authors:** C. Cazorla, M. Enea, F. Lucht, D. Raoult

**Affiliations:** * Centre Hospitalier Universitaire de Saint Etienne, Saint Etienne, France; † Université de la Méditerranée, Marseille, France

**Keywords:** *Ricketssia slovaca*, culture, letter

**To the Editor:** *Rickettsia slovaca* is a bacterium that infects *Dermacentor marginatus* ticks in central and western Europe. First detected in ticks, the bacterium was subsequently identified with genomic amplification by using polymerase chain reaction (PCR) followed by sequencing in a skin biopsy from a French patient (*1*). We describe the first isolation of the organism from a patient.

A 79-year-old woman from St. Etienne, France, found a tick on the parietal area of her scalp 6 days after she returned from a trip to rural southern Burgundy. The tick was removed and subsequently identified as an adult female *D. marginatus* tick. The patient saw a physician 1 day later for low-grade fever (38°C) and myalgia. She was given amoxicillin (3 g once a day), but the fever worsened. She was examined at University Hospital on September 24, 2001, 4 days later. At that time, the patient had a fever; the site of the tick bite showed a necrotic black lesion surrounded by an erythematous halo 4 cm in diameter. Right cervical lymphadenopathy and a papular rash consisting of 10 pink spots on the thorax and arms were observed. Routine blood tests were within normal ranges but asparate aminotransferase (53 IU; normal <45), creatine phosphokinases (140, normal <120), and lactate dehydrogenases (890, normal <620) were elevated. A skin biopsy from the patient’s scalp, serum, and the tick were sent to Marseille to test for possible rickettsial infection. The patient was treated with doxycycline (200 mg once a day, 15 d), and her condition improved. At a check-up 1 month later, she complained of fatigue and insomnia; 2 months later, she had recovered completely, although alopecia still appeared at the site of the tick bite.

*R. slovaca* was demonstrated in the tick and the biopsy by using PCR with primers derived from the citrate synthase and the rOmpA genes as previously reported (*2*). *R. slovaca* was found in human embryonic lung cells (*2*), 3 days after the cells were injected with the skin-biopsied material. Seroconversion, determined by indirect immunofluorescence, occurred with titers to both *R. slovaca* and *R. conorii* of <1/8 and 1/128 in acute- and convalescent-phase sera (sampled 2 months later), respectively.

*R. slovaca*, first identified in dermacentor ticks from Slovakia, has subsequently been found in both *D. marginatus* and *D. reticulatus* in France, Switzerland, Portugal, Spain, Armenia, and Germany (*3*). Since the first human infections with *R. slovaca* were reported, patients with similar clinical signs have been observed in France and Hungary (*4*). Some of these cases have been confirmed by PCR and others by serology (*3*), although serologic titers are frequently low and show cross-reactions with other *Rickettsiae*. We have described the isolation of *R. slovaca* from a patient, which provides the first definitive evidence that *R. slovaca* is a human pathogen. Clinical signs of infection consist of a skin lesion at the site of a tick bite on the scalp (often a dermacentor tick) and regional lymphadenopathy that may be painful. Fever and rash develop subsequently, and the acute disease can be followed by fatigue and residual alopecia at the bite site. The disease may be prevalent within the distribution range of *D. marginatus* and *D. reticulatis* in southern, western, and central Europe. This new spotted rickettsiosis should be added to the list of recognized rickettsial diseases, mainly those caused by *R. africae* (*5*), *R. felis* (*6*) and *R. mongolotimonae* (*7*,*8*).
